# Investigation of Human Cancers for Retrovirus by Low-Stringency Target Enrichment and High-Throughput Sequencing

**DOI:** 10.1038/srep13201

**Published:** 2015-08-19

**Authors:** Lasse Vinner, Tobias Mourier, Jens Friis-Nielsen, Robert Gniadecki, Karen Dybkaer, Jacob Rosenberg, Jill Levin Langhoff, David Flores Santa Cruz, Jannik Fonager, Jose M. G. Izarzugaza, Ramneek Gupta, Thomas Sicheritz-Ponten, Søren Brunak, Eske Willerslev, Lars Peter Nielsen, Anders Johannes Hansen

**Affiliations:** 1 Centre for GeoGenetics Natural History Museum, University of Copenhagen Østervoldgade 5-7, 1350 Copenhagen K, Denmark; 2 Center for Biological Sequence Analysis. Department of Systems Biology, Technical University of Denmark, Building 208, 2800 Lyngby, Denmark; 3 Department of Dermatology D92, Bispebjerg Hospital, Bispebjerg Bakke 23, 2400 N, Copenhagen, Denmark; 4 Department of Heamatology Aalborg University Hospital, Sdr. Skovvej 15, 9000 Aalborg, Denmark; 5 Department of Surgery Herlev Hospital, Herlev Ringvej 75, 2730, Herlev, Denmark; 6 Department of Pathology Herlev hospital, Herlev Ringvej 75, 2730, Herlev, Denmark; 7 Department of Microbiological Diagnostics and Virology, Statens Serum Institut Artillerivej 5, 2300 Copenhagen S, Denmark; 8 Department of Epidemiology Research, Statens Serum Institut Artillerivej 5, 2300 Copenhagen S, Denmark; 9 Health Sciences and Technology Aalborg University Fredrik Bajers Vej 7D2, 9220 Aalborg, Denmark

## Abstract

Although nearly one fifth of all human cancers have an infectious aetiology, the causes for the majority of cancers remain unexplained. Despite the enormous data output from high-throughput shotgun sequencing, viral DNA in a clinical sample typically constitutes a proportion of host DNA that is too small to be detected. Sequence variation among virus genomes complicates application of sequence-specific, and highly sensitive, PCR methods. Therefore, we aimed to develop and characterize a method that permits sensitive detection of sequences despite considerable variation. We demonstrate that our low-stringency in-solution hybridization method enables detection of <100 viral copies. Furthermore, distantly related proviral sequences may be enriched by orders of magnitude, enabling discovery of hitherto unknown viral sequences by high-throughput sequencing. The sensitivity was sufficient to detect retroviral sequences in clinical samples. We used this method to conduct an investigation for novel retrovirus in samples from three cancer types. In accordance with recent studies our investigation revealed no retroviral infections in human B-cell lymphoma cells, cutaneous T-cell lymphoma or colorectal cancer biopsies. Nonetheless, our generally applicable method makes sensitive detection possible and permits sequencing of distantly related sequences from complex material.

It is estimated that almost one fifth (18.6%) of all cancers in humans have an underlying infectious aetiology^1^. Among these are important viral infections such as Epstein-Barr virus (EBV), hepatitis B and C virus (HBV and HCV, respectively), and most notably human papillomaviruses (HPV). Retroviruses are also involved in human cancers. Human T-lymphotropic virus type 1 (HTLV-1) causes adult T-cell leukaemia/lymphoma, whereas HTLV-2 has been associated with cases of myelopathy but its relation to cancer remains controversial^2^. Several animal retroviruses likewise cause lymphoma, leukaemia or other lympho-proliferative diseases in birds and mammals^3^. It is conceivable that undiscovered retroviruses might be involved in human cancers or lympho-proliferative diseases. The detection of such viruses is challenging.

Sequence variation is one of the main challenges in virus discovery. Extensive variation in nucleotide sequence complicates virus family classification (e.g. *Picornaviridae*). This applies also to most retroviruses that show considerable sequence variability. HIV-1 inter-subtype variation exceeds 35% in the variable *env* gene^4^ and >10% in the relatively conserved *gag* and *pol* genes^5^. Additional sequence variation between virus species is contributed by the presence of additional species-specific genes.

The proportion of viral nucleic acids in a cancer sample is usually very small as compared to host-derived genetic material. Firstly, retroviral genomes rarely exceed 10–12 kb, and hence constitute a minor fraction of the genome of the infected host cell. Secondly, the infected cell type may constitute only a small fraction of the sample, and thirdly, the infected cells may contain a relatively low number of viral genome copies. In Kaposi’s sarcoma lesions the Human Herpes virus 8-positive spindle cells constitute only a fraction of all atypical cells. Likewise, retrovirus genomes in humans (e.g. HTLV-1 or HIV-1) are typically present in infected individuals as single integrated proviral copies in minor fractions of nucleated cells in peripheral blood.

Sensitive detection of unknown viral sequences can be undertaken by high-throughput sequence-independent shotgun sequencing. However, because of the quantitative disproportion between viral and host genomic material, no more than a few viral sequence reads can be expected per million reads from host DNA. The proportion of viral nucleic acids can be greatly enriched by mechanical and enzymatic procedures that reduce the host genetic material^6^,^7^,^8^ combined with (random) amplification of the capsid-protected viral metagenome^9^,^10^. These methods are not feasible for investigation of integrated proviral DNA or episomal latent viral nucleic acids. Instead, target enrichment by hybridization (or target capture) can be performed; either in-solution or on solid-surface arrays or beads.

Target capture has been applied to diagnostics^11^, array analysis of virus^12^,^13^, or SNP analysis^14^, and used for enrichment of high-throughput sequencing libraries^15^,^16^,^17^. Most methods are dependent on stringent reaction conditions for discrimination between correct target and competing irrelevant sequences with varying similarity. Kane *et al.* established that cross hybridization may happen if nucleotide sequence similarity exceeds around 75%^18^, unless carefully controlled^19^. Matching stretches of as little as 12–15 complementary nucleotides are sufficient to mediate unspecific cross-hybridization of 50-bp oligonucleotides^18^,^19^. The risk of cross-hybridization has prompted researchers and manufacturers to maximize stringency during capture, including tightly controlled reaction conditions involving denaturing compounds (e.g. formamide) and optimized temperatures.

Where sequence variation poses a challenge, highly specific methods, such as PCR, may be applied with low stringency. For example, lowering of the annealing temperature, inclusion of promiscuously annealing nucleotides (e.g. inosine), or increased MgCl_2_ concentrations may decrease PCR specificity and enable amplification of variant sequences. Capture probes are longer than standard PCR primers and have been used for solid-surface microarray detection of viral sequences^13^. Similarly, we hypothesized that the conditions in capture enrichment may be tweaked to allow sufficient cross-hybridization to recover genomic material from unknown viruses from sequencing libraries prepared from patient sample material.

We have explored the use of in-solution target enrichment in virus discovery using DNA capture probes. We have compared shotgun Illumina library sequencing of integrated provirus with target-enriched library sequencing. Our results showed enrichment of control material with varying sequence similarity to bait, redefining the lower limit of detection and improving the sequence coverage. The developed method was used to conduct an investigation of samples from the clinically important cancer types; colon cancer, T- and B-cell lymphoma. We chose to investigate human lymphoma samples also because this kind of cancer in various animals may be caused by retrovirus infection. Our low-stringency capture method shows important potential in pathogen discovery in complex sample material containing small proportions of distantly related sequences.

## Results

### Probe design

From GenBank we selected genomic sequences from 118 exogenous retroviruses, which are associated with cancer in a vertebrate animal species or in humans (Supplementary Table 1). For certain viral species, sequences from several strains were collected that represent sequence variation. The collected genomic sequences represent viruses from all major branches in the retrovirus phylogeny^20^. The selected sequences (0.87 Mb) were used as templates for custom design and synthesis of probes. Probe lengths ranged between 60–94 nucleotides (average ± st.dev. = 73.6 ± 4.17). Mapping of the hybridization probe sequences to the selected retrovirus genome sequences revealed extensive tiling of probes resulting in full coverage except for short stretches around ambiguous positions in the reference genomes.

Initial testing of target enrichment was performed on control material consisting of human genomic DNA (gDNA) containing 1.2 × 10^3^ copies of HIV-1_Bx08_ provirus DNA per μg, matching the probes perfectly. The enrichment of target (represented by HIV-1*gag*) as well as loss of non-targeted autosomal DNA sequences (B2m) was measured using quantitative PCR.

**Figure 1:**
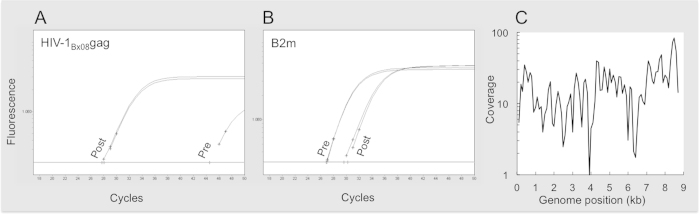
Analysis of target enrichment. Amplification plots from qPCR analysis of HIV-1*gag* target enrichment (**A**) or loss of non-target B2m DNA (**B**) pre- or post capture. Analysis of reference genome (AY713411) coverage of unique reads-pairs from captured library (**C**).

Prior to enrichment, HIV-1*gag* was detectable in library DNA in one of two replicates with a high C_T_-value (~44), indicating a low number of DNA fragments containing the HIV-1*gag* sequence. In contrast, a comparable amount of target-enriched library DNA contained substantially higher quantities of HIV-1*gag* in both replicates (Fig. 1A). A modest decrease was seen for the non-enriched human B2m target after enrichment (Fig. 1B). The results encouraged us to use quantitative PCR to estimate target enrichment and de-selection of non-target DNA fragments in optimization experiments, prior to sequencing.

Target enrichment was determined by comparing Illumina sequencing data obtained before and after capture target enrichment. Prior to capture, shotgun sequencing revealed no sign of HIV-1 provirus in the library, as no reads (of total reads >27 × 10^6^) mapped to the HIV-1_Bx08_ reference genome. In contrast, in two experiments we found 87 or 225 unique read pairs, respectively, mapping to the HIV-1_Bx08_ reference genome in capture-enriched library DNA (Table 1) in dataset of comparable size (1.5 − 2.0 × 10^7^ unique reads). The level of detection was sufficient to achieve full coverage (>22×) of the HIV-1_Bx08_ genome (Fig. 1C). In the negative control libraries (non-infected donor PBMC DNA), HIV-1 reads were not detected (Table 1).

### Target enrichment increases sensitivity of provirus detection

We investigated target capture of varying quantities of proviral HIV-1 in gDNA from blood donors (1 μg/reaction). It was determined that the HIV-1 targets shared 100% (HIV-1_Bx08_) or 86% (HIV-1_CC0030_) sequence similarity to the bait (Table 1).

Shotgun sequencing resulted in no read pairs mapping to the HIV-1 reference genome at quantities <9.1 × 10^3^ HIV-1 copies per reaction. In material containing 1.4 × 10^5^ copies of provirus, 3 to 10 unique read pairs were detected corresponding to an on-target ratio of 0.6–14.2 per million reads (Table 1). Considering the genome size of proviral HIV-1 and the applied sequencing depth, this is consistent with the expected numbers (0–1 read) mapping to HIV-1.

**Figure 2:**
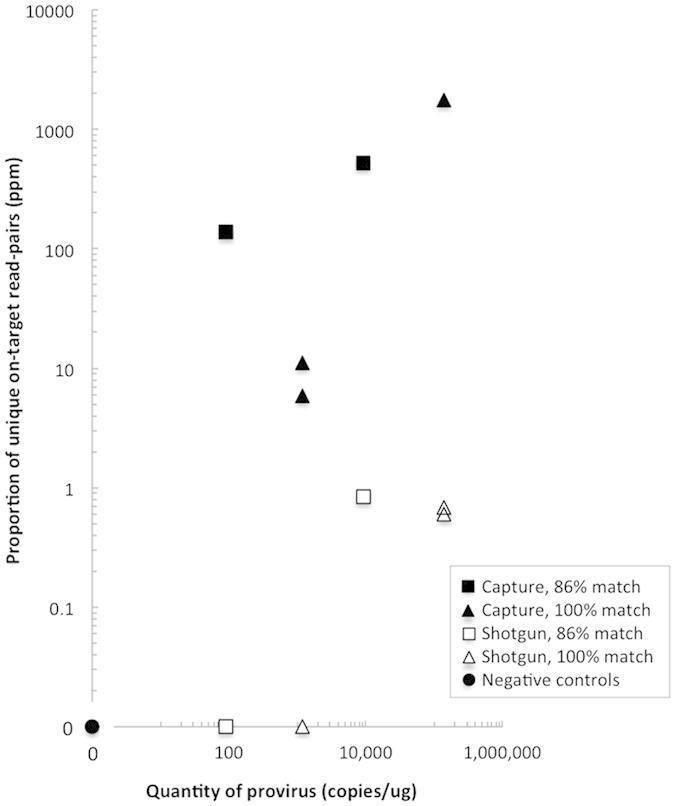
Relationship between input of viral target and proportion of unique reads mapping to HIV-1 target for captured (solid) or non-captured libraries (open). Targets and probes showed 86% (squares) or 100% (triangles) sequence similarity. Captured negative control samples are included (round). Results are expressed as on-target unique reads per million of total reads (ppm). ND: None detected.

In target-enriched libraries ≥18 unique read pairs were detected that map to the reference genome at quantities down to 9.1 × 10^1^ copies/μg DNA (Table 1). As seen in Fig. 2, there is a clear relationship between the quantity of provirus and the proportion of unique read pairs in sequenced target-enriched libraries.

When comparing target enrichment with shotgun sequencing we found on-target ratios after target enrichment to be 1–3 orders of magnitudes higher (Fig. 2). Furthermore, target capture resulted in increased sensitivity of provirus detection at the present sequencing depth (Table 1).

### Viral target capture at low stringency conditions

The in-solution capture format permits lowering of the stringency of hybridization by decreasing the hybridization temperature or adjusting the formamide concentration. Both alternatives were tested using human gDNA containing known quantities of proviral HIV-1_CC30_ DNA only 86% similar to bait.

**Figure 3:**
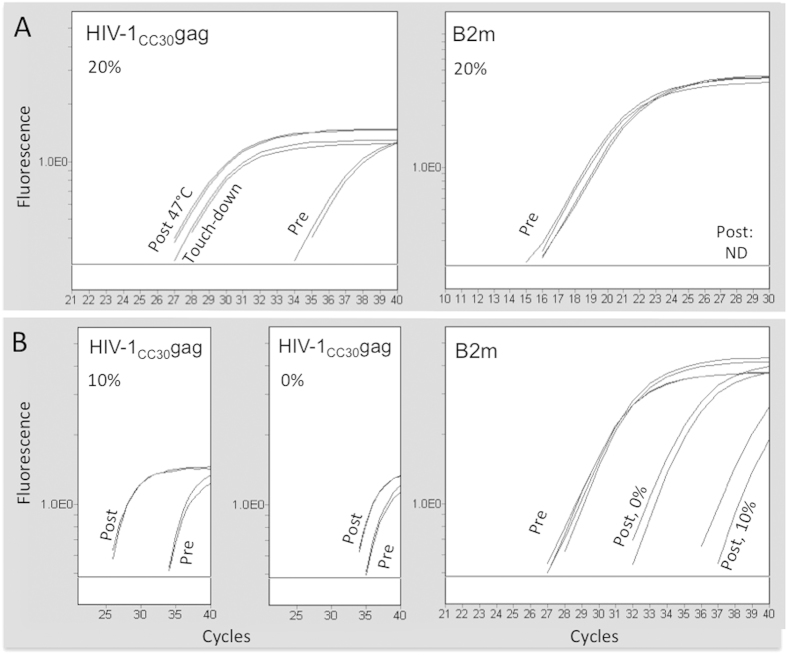
Quantitative PCR analysis of sequencing libraries. Relative enrichment of target (HIV-1_CC30gag_) and loss of non-target target (B2m) were measured by qPCR pre- and post-capture. Capture was performed (**A**) using 20% formamide and different temperatures (47 °C or touch-down), or (**B**) at reduced formamide concentrations (10% or 0% as indicated). All samples were performed in 2 or 4 technical replicates. ND: Not detected.

Inspired by Mason *et al.*^21^, we compared in-solution hybridization at the recommended reaction temperature with gradually decreasing temperatures. In libraries before and after capture, qPCR analysis indicated comparable enrichment of relevant target (HIV-1*gag*) and comparable lessening (to C_T_>30) of non-targeted autosomal (B2m) DNA fragments at both conditions (Fig. 3A).

Quantitative PCR analysis also indicated that capture at 47 °C, using 20% or 10% formamide, also resulted in comparable enrichment of relevant targets. In contrast, 0% formamide reduced the enrichment of relevant target while increasing the recovery of non-target sequences (Fig. 3B).

**Figure 4:**
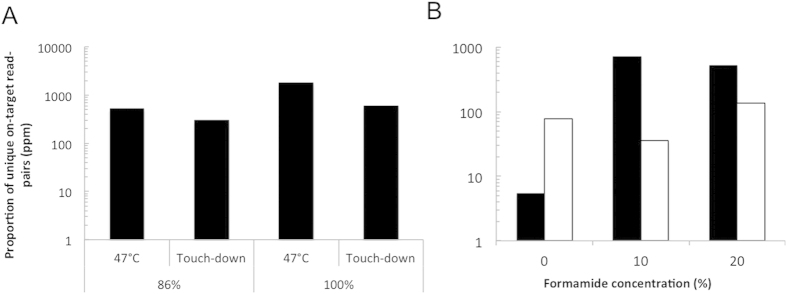
Enrichment of on-target sequences with lowered stringency conditions. (**A**) Comparing hybridization temperature conditions for virus with indicated similarity to bait. (**B**) Titration of formamide using gDNA with 9 × 10^3^ copies/ug (black) or 91 copies/ug of HIV-1 (white) 86% similar to bait.

Illumina sequencing corroborated these qPCR results. For sub-optimally matching target, or matching target we found no indications that touch-down temperatures improved on-target ratios of reads mapping to reference sequence (Fig. 4A). The reduction of formamide concentration from 20% to 10% seemed to permit an increased proportion of unique reads in the sample with 9.1 × 10^3^ copies/reaction (Fig. 4B).

### Enrichment of distantly related sequences using low stringency capture

Our overall aim was to capture distantly related exogenous retroviral sequences in complex samples from humans. Even short complementary regions may mediate hybridization between capture probes and targets^18^,^19^ within the host genome e.g. orthologous genes and human endogenous retrovirus (HERV) sequences. Consequently, we investigated the extent of such cross-hybridization in our samples, potentially resulting from unwanted capture of short complementary host genome sequences.

**Figure 5:**
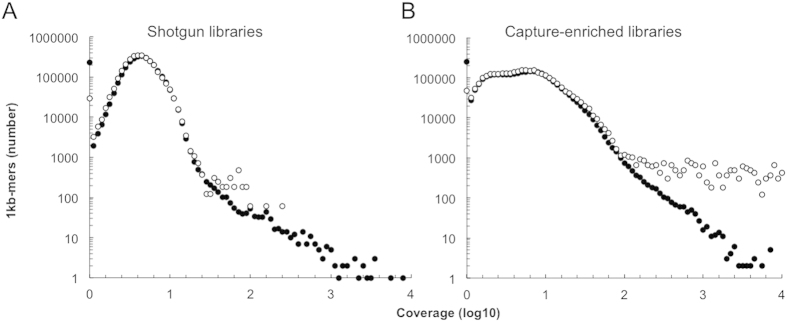
Analysis of similarity between probes and host genome. The normalized number of 1 kb-mers is shown from the human reference genome (Hg19) that contains ≥1 motif of ≥25 bp nucleotides with perfect identity to any of the capture probes (○) or without such motif(s) (•). Data from all sequencing shotgun sequencing experiments (**A**) and capture-enriched experiments (**B**) are merged.

We divided the host genome (hg19) into two groups of 1 kb bins; those containing at least one stretch of ≥25 nucleotides with perfect identity to the sequences of the capture probes, and those without. We analysed the sequence coverage in these host genome regions obtained after shotgun- or capture-enriched sequencing (Fig. 5).

After capture enrichment a higher number of regions with 25-mer probe similarity were sequenced with coverage greater than approximately 100 (Fig. 5B). Shotgun sequencing did not show the same bias (Fig. 5A). The phenomenon was most pronounced in libraries captured in 10% formamide. In contrast, touchdown temperature conditions did not increase this cross-hybridization. Our results showed that we selectively detected the regions in the host genome that contained regions (≥25 bp) identical to probes. These results corroborated the previous analyses, and indicate that identity in relatively short regions is sufficient for capture enrichment of 100–1000 bp DNA fragments; particularly at low stringency conditions.

### Capture of distantly related PERVs

**Figure 6:**
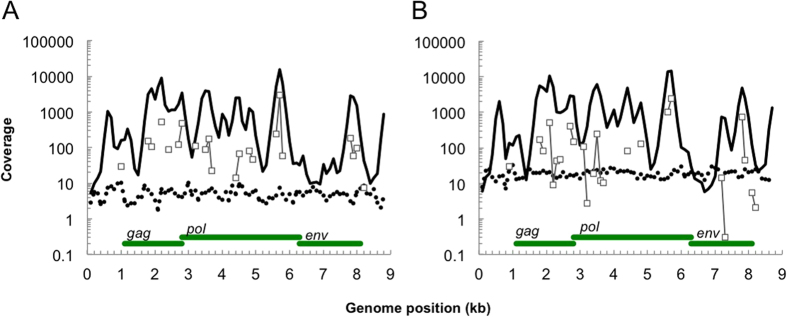
Low stringency target enrichment of PERV-A and PERV-B genomes from infected HEK293 cell lines. Coverage of PERV-A (**A**) or PERV-B (**B**) genomes after shotgun sequencing (dashed) or low stringency target enrichment (solid lines). The coverage of 25-mer matching probe motifs are shown in (grey □). Location is indicated of the *gag*, *pol* and *env* genes.

To further explore the concept of intended cross-hybridization, we tested to what extent distantly related proviral sequences were enriched by capture. The extent of capture was investigated in human embryonic kidney (HEK293 cells) containing multiple copies per cell of proviral porcine endogenous retrovirus (PERV) DNA. The pairwise similarity of any capture probe to the PERV genomes was <91%. Approximately 2% of the capture probes showed ≥60% overall similarity to PERV genomes in regions of *gag*, *pol* and *env*. Predictably, we achieved highest coverage in these regions upon capture (Fig. 6).

With shotgun sequencing the proportion of unique on-target reads was 159 or 516 per million reads (ppm). This proportion translates into 3× or 12.8× coverage, respectively, for proviral PERV-A or PERV-B DNA, with a sequencing depth of 65 × 10^6^ or 138 × 10^6^ reads/sample (Fig. 6). As would ideally be expected from shotgun sequencing, the coverage was relatively evenly distributed over the provirus genomes. The obvious finding is that proviral sequences may be detected with sufficient sequencing depth and virus load.

Moreover, target enrichment by low stringency capture increased the average on-target proportion 174 and 252-fold, respectively, yielding an average sequence coverage of 865 or 949 (Fig. 6). As predicted, coverage tended to peak in regions of the genome with relatively high similarity to the capture probes. Indeed regions with high coverage after capture also contained ≥25-mer motifs identical to capture probes (Fig. 6). All capture probes with such similarity were designed specifically for 17 non-PERV gamma retrovirus reference genomes (see Supplementary Fig. S1 online). Importantly, the reads mapping to the PERV reference sequences did not map elsewhere to annotated genomic HERV sequences (see below), reducing the possibility that reads derived from HERVs. In summary, target (proviral) sequences can be enriched by distantly related probe sequences at low stringency conditions.

As a relatively large proportion of the human genome consists of HERV sequences, enrichment of HERV sequences could be expected. Among the HERV families, the HERV-K is the biologically most active. In some contexts, members of the HERV-K family produce viral particles^22^. Cloned HERV-K113 proviral DNA produces particles in baculovirus-infected Sf9 cells^23^. Because HERV-K particles are also seen in germ cell cancer and melanoma^24^ a role in disease has been suspected. HERV-K113 is distantly related to the beta-retroviruses jaagsiekte sheep retrovirus (JSRV) and mouse mammary tumor virus (MMTV), hence 0.041% of the capture probes map with ≥60% similarity to three distinct regions of the HERV-K113 genome (see Supplementary Fig. 2 online). Consequently, although HERV-K113 is readily detected by shotgun sequencing of HEK293 cells, a small enrichment of HERV-K113 was seen but only in regions with similarity to probes. These results indicate the limits for cross-hybridization.

### HIV-1 samples

We investigated peripheral blood from HIV-1-infected individuals (subtype A or B) to demonstrate that the sensitivity of the method is sufficient for clinically relevant samples. For both samples, HIV-1 reads were detected (0.48–0.74 ppm) after low-stringency capture, which is lower than the captured positive control samples (Fig. 2), suggesting a limited DNA viral load. No viral reads were detected in the shotgun sequencing dataset despite sequencing more than 67 × 10^6^ reads per sample (see Supplementary table 2 online). These results demonstrate the method has sufficient sensitivity for detection of proviral DNA in typical clinical sample material.

### Capture enrichment of retroviral DNA sequences from cancer samples

Because our capture method enriches for retroviral sequences that may be only distantly related, it has applications in virus discovery. A number of retrovirus species are known causes of cancer, such as lympho-proliferative diseases, in animals and humans^3^,^25^. Therefore, we conducted sequence analysis of capture-enriched libraries from B-cell lymphoma cell lines, T-cell lymphoma. As colorectal cancer is an abundant cancer type, such samples were also investigated the presence of reads showing similarity to known vertebrate viruses (Table 2, and Supplementary Table 3).

In capture-enriched libraries prepared from gDNA extracts (34.2–191.1 million trimmed reads per library), many read pairs showed apparent similarity to known acutely transforming retroviruses, such as Abelson murine leukaemia virus, Rous sarcoma virus and others. Further analysis of the location of the reads showed that these reads mapped within the viral oncogenes (e.g. *v-abl* or *v-src*). In all cases BLASTn^26^ analysis of reads or contiguous sequences mapping to viral oncogenes showed higher similarity to the orthologous sequences in the human genome. Thus, as expected, the probes included for oncogenic retroviruses enrich for human orthologous sequences as well. Importantly since initial automated digital subtraction against hg19 required ≥97% sequence identity, some reads with less sequence similarity were not digitally subtracted.

In addition, we conducted shotgun sequencing of DNA libraries (Table 2). As expected, the reads mapping within cellular orthologs to viral oncogenes, were orders of magnitude less abundant in these datasets. Some shotgun-sequenced gDNA libraries from fully transformed B-lymphoma cells (n = 12), biopsies containing cutaneous T-cell lymphoma (n = 6) and biopsies from patients with colorectal cancer (n = 13), contained low numbers of read pairs (often ≤5), mapping to reference genomes of viruses commonly found in humans (e.g. parvovirus B19, herpesviruses) (Table 2). Such findings were not investigated further, as they are all readily explained as random findings not related to the cancer. In one sample (CGG_5_000274), 3223 read pairs mapped across the human herpesvirus type 6A (HHV-6A) reference genome (GenBank NC_001664.2). The proportion of read pairs mapping to HHV-6A or hg19 corresponded to the relative sizes of the HHV-6A and the human diploid genome, suggesting that the sample originated from a patient with a chromosomally integrated copy of HHV-6A. Integrated HHV-6 typically results in high viral loads in all sites^27^, but has no known association to cancer. The results emphasize that shotgun sequencing is sufficiently sensitive to detect viral genomes only when present in unusually high proportions of complex sample material.

### Capture of mRNA libraries

In all RNA libraries we found read pairs mapping to the parvovirus-like hybrid virus (PHV), reference genome (GenBank NC_022089.1). In the digitally subtracted shotgun libraries (n = 16), frequencies ranged between 1.4–655.5 ppm PHV reads. As described by Naccache *et al.*, PHV was found to be a contaminant of the RNA purification kits^28^. PHV sequences were not eliminated entirely in capture-enriched libraries, although the proportions of PHV seemed to be smaller in these (3.6–7.2-fold, n = 5). Likewise, all shotgun-sequenced RNA libraries contained read pairs mapping to avian retrovirus reference genomes (e.g. 11.2–1005.6 ppm, median = 139.5, n = 16, post digital subtraction for AY350569). These are likely derived from residual avian retrovirus genomic material in the ScriptSeq reverse transcriptase enzyme. Subsequent enrichment by retrovirus-specific probes increases the frequency of these sequences by several orders of magnitude.

Analysis of DNA and RNA libraries in this study uncovered no novel viruses involved in cancer. The presence of PHV-like sequences and avian retroviral sequences in RNA kits or reagents may complicate virus discovery.

## Discussion

In the present experiments we have cast a wide-ranging net to capture and enrich sequencing libraries for unknown exogenous retroviral DNA sequences in potentially very low proportions of non-target DNA. Thus, our application of target capture is fundamentally different from other applications where the target may be several megabases of abundant sequence^15^,^16^,^29^,^30^,^31^, or degraded ancient DNA in complex sample material^15^,^32^. For this investigation, selection of reference genomes included known exogenous virus species. As the purpose of the study was to screen cancer material for retrovirus, we accepted that the probes may tend to over-represent viral species already abundant in the sequence database. Analysis of DNA and RNA libraries in this study uncovered no new retroviruses involved in cancer.

One of the challenges to high-throughput sequencing in pathogen discovery is to detect rare targets in a vast excess of host DNA^33^. To our knowledge only two studies have used capture enriched high-throughput sequencing of viral targets; both for known and invariable sequences. Depledge *et al.* capture-enriched sequencing libraries for low titer episomal herpesvirus from a vast excess of host genetic material using RNA probes^17^. Likewise, integrated Merkel cell polyomavirus sequences were enriched by target capture from formalin-fixed paraffin-embedded tumour samples using PCR amplicons as bait^34^. We have detected different subtypes of proviral HIV-1 sequences in clinical samples upon capture. We found no indications of artefact contaminating reads in control samples, suggesting that our method permits virus discovery for low titre integrated viral targets with unknown, but related, sequences in an abundant background. This resembles capture enrichment of endogenous ancient DNA from complex material^32^.

A number of new viruses have been identified using different means of enrichment, including mechanical and enzymatic depletion of host genomic material in combination with subsequent randomly primed PCR^9^,^10^,^35^,^36^. Whereas these methods have been successful in detection of virions from humans and animals, proviral DNA can remain undetected by random PCR^37^. Integrated-, latent episomal- and virion-associated viral sequences may be enriched from libraries by capture^34^.

Considerable sequence variation exists for both DNA and RNA viruses. A very divergent type of human papilloma virus (HPV) was described showing only 61% similarity to other HPV types^38^. Among the RNA viruses, the inter-subtype genetic distances between HCV strains exceeds 30%^39^. Retroviruses (e.g. HIV-1) are also highly variable^4^,^5^. Therefore the sequence-specific methods, such as high-throughput multiplex qPCR^40^ or microarrays^11^, may be inadequate for discovery of novel viruses. Low stringency capture enrichment increases the possibilities of detecting viral DNA and RNA from unknown virus genotypes or species^13^. By enrichment of the distantly related PERV sequences, we demonstrate that the stringency, or specificity, in the experiments is low enough to allow enrichment of new virus *species* among the variable retroviruses. In a similar way, low stringency conditions were originally used for identification of certain HERVs using probes derived from non-human retroviruses^41^,^42^. Other studies have used touch-down temperature conditions to enrich mitochondrial DNA or chromosomal sequences from related animal species with >60% sequence similarity^21^,^43^. Similarly, sub-stringent temperatures were used to allow cross-species hybridization of short DNA fragments in historical material^32^,^44^. In our experiments the reduced formamide concentration (10%) or a 5 °C gradual reduction of temperature both relaxed the hybridization stringency which is comparable with other studies^21^,^43^.

We selectively detected genomic regions in virus- and in the host genomes that match the bait. Data analysis generated similar results using two different criteria; ≥60% overall similarity^43^ or perfectly matching ≥25 bp motifs. The 25 bp motif criterion more accurately explained our results (Figs 5 and 6). This is consistent with the notion that a continuous stretch of annealing DNA is advantageous to the formation of a helical conformation which is stable during hybridization, and also in good agreement with results from a microarray study^13^.

The sensitivity of the methods used in virus discovery is rarely assessed. In capture-enriched libraries, we detected as few as 91 copies of HIV-1 proviral DNA per μg gDNA (Table 1). This corresponds to a lower detection limit of approximately <700 viral copy per 1 × 10^6^ cells, which is comparable to the DNA load in other human retroviral infections^45^. Inclusion of samples from HIV-1-infected individuals substantiates that the sensitivity of the method is sufficient for relevant clinical material, although in HIV-1 elite controllers the DNA load may be only 10–80 copies per million cells^46^.

From the numbers of on-target reads obtained by shotgun sequencing (Table 1) we estimate that >1 × 10^8^ reads per library would probably be required to reach the same level of sensitivity with shotgun sequencing. The level of sensitivity is comparable to studies with random-primed PCR^10^,^37^. In contrast the detection limit in microarray was >1 × 10^4^ viral DNA copies per reaction^13^.

While the cost of sequencing continues to decrease, capture probe libraries remains to be a considerable expense. The cost per sample may be reduced by immortalized probe libraries^47^ or by multiplexing of samples^48^. In virus discovery application, where the target is unknown (and not known to be present), enrichment improves the lower detection level. Our comparison of the proportion of unique on-target reads pre- and post-enrichment was commonly 1–3 orders of magnitude (range 18–2948 fold), which is comparable to other studies using a single round of capture of various targets^16^,^43^,^49^,^50^. The impact on improved cost and efficiency of downstream bioinformatics analysis is not to be underestimated. As sequencing depth and data volumes are rapidly improving, the bioinformatics is more tractable with enriched sequencing data that is orders of magnitude less voluminous than untargeted sequencing. More importantly, pre-enriched sequencing also helps with matching reads to targets where borderline hits can be more unambiguously defined. Benefits of this would be seen especially in detection of unknown/novel viruses where reads would be imperfectly aligned to known viral material.

Seven different viruses are currently known to cause cancer in humans. HTLV-1 is the aetiology of adult T-cell leukaemia^25^ and other retroviruses are involved in various animal lympho-proliferative diseases^3^. Therefore, we included human lymphoma types in our investigation. We also investigated colon cancer samples for traces of retroviral infection as evidenced by proviral DNA and, to a lesser extent, viral RNA. Here we show for the first time the results of screening for unknown expressed or non-expressed-, integrated-, or latent episomal viral DNA in human cancer using massive parallel sequencing. The method complements other methods like sequence-independent PCR on material previously enriched for virions (e.g.^10^), and array detection^12^,^13^.

Our capture-enriched or shotgun libraries revealed evidence of other known viral infections but not for retroviral infections in human cancer samples. These results are in good agreement with recent analyses of shotgun RNA sequencing data from thousands of samples from various cancer types, but not including lymphoma samples^51^,^52^.

The probes mediated enrichment for host (human) genome sequences encoding cellular orthologs to retroviral oncogenes (e.g. c-*abl* and c-*src*). The bias was most pronounced in libraries captured at 10% formamide, whereas touchdown temperature conditions, in our experiments, were ineffective in improving cross-hybridization. Enrichment of cellular ortholog sequences is an acceptable by-product, and servers as an internal control, in the search for new pathogens. A minor concern may be that an abundant cellular ortholog may impair sensitive detection of a retroviral counterpart if rare.

Although intended cross hybridization may not result in full coverage of a viral pathogen, even a very limited coverage may allow subsequent targeted research^53^. The probability of identifying new viruses in cancer is likely to be lower than in samples from patients with acute febrile illness. The selection of probes determines the broadness of the analysis. For this study, probes covered genome sequences from 118 retroviruses associated with cancer. Our results show that distantly related sequences are captured (incl. virus species in control material, reagent contaminants or cellular orthologs). Likewise, we believe that unknown but distantly related viral sequences would have been detected if present in concentrations higher than one viral copy per 1000 host cells.

Our method has several advantages; firstly, it allows detection of episomal or integrated DNA or RNA^54^, secondly, it offers sensitivity superior to microarray technology^13^ and comparable to that of random PCR^10^, and thirdly, it is compatible with standard protocols for Illumina sequencing platforms and requires only standard laboratory equipment^54^. Finally, it offers a flexible format of probes (i.e. target size, tiling) that can be individually adapted to different applications. Applications of the low stringency target-enriched sequencing are numerous within several fields of research in which sequences are unknown or variable. Targeted enrichment reduces the cost of sequencing and consequently enables increased number of samples and/or sequencing depth. The method may be an alternative to multiplex PCR or long-range PCR to study gene families or inter-species genetic markers of (distantly) related species. In pathogen discovery enrichment with probes similar to any group of pathogens (e.g. virus, bacteria or fungi) can provide a first handle on the unknown related species. Furthermore, it may be possible to improve enrichment of endogenous DNA cross-species in ancient DNA studies^32^,^49^, or within selected taxa in environmental DNA studies^55^.

In this study we demonstrate that retrovirus are sensitively detected by high-throughput sequencing after enrichment with hybridisation probes, based on distantly related retroviral sequences. In accordance with recent studies on different cancers, our investigation of human B-cell lymphoma cells, cutaneous T-cell lymphoma or colorectal cancer biopsies revealed no retroviral infections associated with cancer.

## Methods

### Ethics statement

The following two ethical boards reviewed the protocol for the present study: The Regional Committee on Health Research Ethics (Case No. H-2-2012-FSP2) and the National Committee on Health Research Ethics (Case No. 1304226). Both review boards waivered the requirement for informed consent, in accordance with national legislation (Sundhedsloven), as the study design included only samples anonymized at collection (at Department of Surgery, Herlev Hospital, Department of Dermatology Bispebjerg hospital or Department of Haematology, Aalborg University Hospital). Sample material was obtained from cancerous tissue already removed during treatment of patients, by JLL, RG or KD, respectively. Dataset depleted for reads mapping to human genome are deposited at Sequence Read Archive.

### Cells and patient samples

Human gDNA containing proviral HIV-1 DNA of isolates Bx08 (GenBank AY713411) and CC0030 (GenBank FJ694791) were generated by standard virus propagation in Phytohemagglutinin-P-stimulated donor peripheral blood mononuclear cells (PBMCs) from blood donors. Frozen whole blood from HIV-1 patients were obtained from Statens Serum Institut.

Human embryonic kidney cell lines (HEK293) infected with porcine endogenous retroviruses (PERV)-A (AJ133817) or PERV-B (AJ133818) were kindly provided by Yasuhiro Takeuchi.

Cryopreserved fully transformed B-cell lymphoma cell lines (RPMI-8226, KMS-12-BM, KMS-12-BM, KMS-12-PE, MOLP-8, MOLP-2, SU-DHL-5, SU-DHL-4, U266, U698M, OCI-Ly8, OCI-Ly7_M, and OCI-Ly3_M) were obtained from Aalborg Hospital, Department of Haematology. Cutaneous T-cell lymphoma biopsies were obtained from Bispebjerg Hospital, Department of Dermatology. Colon cancer needle biopsies were obtained from Herlev Hospital, Department of Surgery. Biopsies were obtained on fresh tissue immediately after surgical resection, by microsurgical dissection in the operating room. The resected bowel was closed at both ends, and a needle biopsy of the tumor was taken through the serosal side of the bowel at the tumour site. In this way it was possible to obtain a tumour biopsy not contaminated by bowel content. A control biopsy was obtained simultaneously from the same site and it underwent microscopy to ensure, that it contained tumour tissue and not only e.g. necrosis.

### Probe design

From GenBank genome sequences were compiled from a total of 118 retroviruses associated with cancer in humans or other vertebrate species (Supplementary Table 1). In instances where several genotypes existed, different variants were included, to represent the existing sequence variation (e.g. ovine nasal tumour virus or HTLV). No identical sequences were included. In 27 cases partial genome sequences were included when no near-full length genomes were available. One near-full length HIV-1 genome (AY713411) was included for control experiments. SeqCap EZ hybridization probes (n = 729,243) were designed and synthesized by Roche NimbleGen (Madison USA).

### Library building

Genomic DNA was extracted from cells, biopsies, or blood according to instructions in QIAamp DNA Mini kit (Qiagen). DNA libraries were prepared from 1 μg of DNA according to the Illumina Truseq DNA protocol (PE-940-2001) or an in-house protocol using the NEBnext E6070 (New England Biolabs) reagents^56^. RNA was extracted from B-lymphoma cells and colon cancer biopsies using mRNA direct Dynabeads (Lifetechnologies) or High Pure Viral RNA kit (Roche), respectively. For lymphoma samples this included ribosomal RNA depletion using RiboZero (Epicentre cat. No. SCL24G) and subsequent purification, using RNeasy MINelute colunms (Qiagen). All RNA libraries were prepared using ScriptSeq Complete Gold Kit (Epicentre) according to manufacturer’s protocol.

### Capture

Target enrichment (capture) reactions were performed with 1 μg of library as described in the SeqCap EZ library SR protocol (Roche NimbleGen). In some hybridization reactions the volume of Hybridization component A (formamide) was replaced with water to reduce final formamide concentrations. In other reactions the hybridization temperature was gradually decreased from 47 °C to 42 °C (1 °C/12 hours).

### Quantitative PCR

The SeqCap EZ library kit (NimbleGen) includes internal PCR controls to monitor capture efficiency. In addition, standard TaqMan qPCR assays were used to monitor enrichment of targets (HIV-1_Bx08_ gag) and deselection of non-targeted gDNA sequences, Beta-2-microglobulin (B2m). Roche LC480 probes master (cat no. 04 707 494001) or Roche LC480 SYBR Green I (cat no. 04707516001) reagents were used in TaqMan or SYBR green qPCRs respectively, according to manufacturer’s instructions using comparable concentrations of template DNA (range 4–28 ng/rxn). All qPCRs were performed on a Roche LC480 instrument. Primers and probes are listed in Supplementary Table 4.

Quantification of HIV-1 provirus in control gDNA was performed at the accredited laboratory at Statens Serum Institut, Department of Virology, according to a procedure published elsewhere^57^.

### Sequencing and data analysis

Pools of Illumina libraries were created using unique sequencing indices. Sequencing was performed on HiSeq 2000 instruments at BGI Europe or the Danish National High-Throughput DNA Sequencing Centre^56^. All sequencing was performed as 100 bp paired-end runs except the two HIV-1 samples, which were 100 bp single-end.

Basic sequence editing, mapping and alignments were performed in Geneious Pro software (ver 5.6.3).

#### Sequence analysis of control samples

Paired-end sequencing reads were cleaned for adapter sequence using AdapterRemoval^58^ which removes adapter sequences and merges overlapping mate sequences. The resulting read pairs and singletons were mapped to the human genome (hg19) and selected viral genomes using BWA^59^. Multiple read pairs/singletons mapping with the same start and end coordinates were considered clonal PCR products and were discarded from further analysis (keeping one representative). Throughout the manuscript the remaining reads are referred to as ‘unique’. Host genome-depleted data from HIV-1-infected individuals were included in BLASTn analysis against reference HIV-1 genomes. Hits exceeding 90% of the read length were counted.

#### Host genome capture analysis

All possible 25-mers from the capture probe sequences were mapped against the human genome. The human genome was divided into non-overlapping 1 kbp windows, and windows overlapping with at least one mapping 25-mer were recorded. This resulted in 50,284 windows with matches to the probe 25-mers and 3,045,381 windows without matches. The average coverage was then compared between the two categories of windows.

#### Digital subtraction and in silico identification of virus

Digital subtraction of human-like read pairs was performed in two steps using the alignment tools BWA^59^ using *aln* default parameters and BLASTn. All read pairs where at least one of the two reads mapped to hg19 were discarded using default alignment parameters. Reads were considered mapped by BWA if they were not assigned SAM flag 4^60^ or if the BLAST alignment was >90% id, 90+ bp and with an e-value <10^−3^. Dataset depleted for reads mapping to human genome are available at Sequence Read Archive (http://www.ncbi.nlm.nih.gov/Traces/sra/) (PRJNA238023). The remaining reads were mapped by BLAST using identical settings to a database consisting of 1333 NCBI RefSeq vertebrate viruses and their 52970 NCBI Genome Neighbors downloaded from the NCBI Viral Genomes Resource. Positive hits were defined as read pairs where both reads mapped within a distance of 1000 bp in a forward-reverse manner.

## Additional Information

**How to cite this article**: Vinner, L. *et al.* Investigation of Human Cancers for Retrovirus by Low-Stringency Target Enrichment and High-Throughput Sequencing. *Sci. Rep.* **5**, 13201; doi: 10.1038/srep13201 (2015).

## Supplementary Material

Supplementary Information

## Figures and Tables

**Table 1:** Summary of sequencing of control samples.

**Library ID**	**Enrichment method**	**Target quantity (Copy number/ug)**	**Sequence similarity to probes (%)**	**Unique on-target reads**	**Sequencing depth (Unique HG pairs)**
11,C	Capture	0	N/A	0	2.7 × 10^7^
11,B	Capture	0	N/A	0	1.8 × 10^7^
10+,C	Capture	1.2 × 10^3^	100	225	2.0 × 10^7^
10+,B	Capture	1.2 × 10^3^	100	87	1.5 × 10^7^
10+,B	—	1.2 × 10^3^	100	0	2.9 × 10^7^
4a	Capture	9.1 × 10^3^	86	2,433§	4.7 × 10^6^
4b	—	9.1 × 10^3^	86	6§	7.1 × 10^6^
C	Capture	9.1 × 10^1^	86	18§	1.3 × 10^5^
C	—	9.1 × 10^1^	86	0§	5.9 × 10^4^
7a	Capture	1.4 × 10^5^	100	13,248	7.5 × 10^6^
7b	—	1.4 × 10^5^	100	3	5.0 × 10^6^
7d	—	1.4 × 10^5^	100	10	1.5 × 10^7^

^§^Compared to reference genome HIV-1_CC0030_ (GenBank FJ694791).

**Table 2:** Summary of cancer sample sequencing data.

**Library (material, method)**	**Cancer type**	**Reads trimmed (range** × **10**^6^**/sample)**	**Reads after digital subtraction (range** × **10**^6^**/sample)**	**Reads mapping to virus**
DNA, capture	T-lymphoma (n = 6)	118.8–191.0	2.0–5.2	HHV6
	B-lymphoma (n = 12)	107.3–218.6	4.2–8.9	
	Colon cancer (n = 6)	34.1–81.7	1.21–2.28	
DNA, shotgun	T-lymphoma (n = 6)	131.7–248.8	3.1–5.9	HHV-6, parvovirus B19
	B-lymphoma (n = 12)	27.5–137.3	0.4–6.1	
	Colon cancer (n = 13)	33.1–369.9	0.4–6.8	
RNA, capture	B-lymphoma (n = 6)	51.4–82.0	8.1–14.1	PHV$, ALV§
RNA, shotgun	B-lymphoma (n = 5)	60.1–117.6	6.0–11.1	PHV$, ALV§
	Colon cancer (n = 11)	7.2–116.4	0.4–10.6	PHV$, ALV§

^$^PHV: Parvo-like hybrid virus. Suspected contaminant from RNA extraction/purification kits^28^.

^§^ALV: Avian leucosisvirus. Suspected contaminant from the ScriptSeq library kit.
