# Supplementary material for: Investigation of Human Cancers for Retrovirus by Low-Stringency Target Enrichment and High-Throughput Sequencing
Source: Sci Rep. 2015 Aug 19;5:13201. doi: 10.1038/srep13201 (PMC4541070; doi:10.1038/srep13201)
Supplement: Supplementary Information [file srep13201-s1.pdf]

Investigation of Human Cancers for Retrovirus by Low-Stringency Target Enrichment and High-Throughput Sequencing

Lasse Vinner, Tobias Mourier, Jens Friis-Nielsen, Robert Gniadecki, Karen Dybkaer, Jacob Rosenberg, Jill Levin Langhoff, David Flores Santa Cruz, Jannik Fonager, Jose MG Izarzugaza, Ramneek Gupta, Thomas Sicheritz-Ponten, Søren Brunak, Eske Willerslev, Lars Peter Nielsen, Anders Johannes Hansen

Supplementary information.

Supplementary Table 1

Accession	Organism	Length (bp)	Taxonomy	Oncogene (ortholog)
AF033812	Abelson murine leukemia virus	5894	Gammaretrovirus	v-abl
V01541	Abelson murine leukemia virus	5893	Gammaretrovirus	v-abl
NC_010820	African green monkey simian foamy virus	13111	Spumavirus	-
DQ174103	Atlantic salmon swim bladder sarcoma virus	10305	Unclassified	-
X00578	Avian carcinoma Mill Hill virus 2	1862	Alpharetrovirus	v-mil
X12707	Avian erythroblastosis virus	6373	Alpharetrovirus	v-erbA, v-erbB
M60582	Avian hemangioma-inducing virus	387	Unclassified	-
S74099	Avian myeloblastosis virus	7226	Alpharetrovirus	v-myb
M11784	Avian myelocytomatosis virus	3621	Alpharetrovirus	v-myc
NC_001866	Avian myelocytomatosis virus	3392	Alpharetrovirus	v-myc
AF001075	Avian sarcoma virus 16	3389	Alpharetrovirus	v-p3k
Y00302	Avian sarcoma virus CT10	2428	Alpharetrovirus	v-crk
AY134750	Bovine foamy virus	12002	Spumavirus	-
AF033818	Bovine leukemia virus	8419	Deltaretrovirus	
AF257515	Bovine leukemia virus	8588	Deltaretrovirus	
EF600696	Bovine leukemia virus	8720	Deltaretrovirus	
FJ914764	Bovine leukemia virus	8585	Deltaretrovirus	
K02120	Bovine leukemia virus	8714	Deltaretrovirus	
J04169	Cas NS-1 murine leukemia virus	2711	Gammaretrovirus	v-cbl
GU356395	Common marmoset foamy virus	11744	Spumavirus	-
AF201902	Equine foamy virus	12035	Spumavirus	-
K02712.1	FBR murine osteosarcoma virus	3811	Gammaretrovirus	v-fos
AB052796	Feline foamy virus	11660	Spumavirus	-
AB052797	Feline foamy virus	11693	Spumavirus	-
AB052798	Feline foamy virus	11757	Spumavirus	-
AJ564745	Feline foamy virus	11658	Spumavirus	-
AJ564746	Feline foamy virus	11658	Spumavirus	-
Y08851	Feline foamy virus	11700	Spumavirus	-
J02088	Feline sarcoma virus	2397	Gammaretrovirus	v-kit
K01643	Feline sarcoma virus	4577	Gammaretrovirus	v-kit
M15805	Feline sarcoma virus	2091	Gammaretrovirus	v-kit
X00255	Feline sarcoma virus	2025	Gammaretrovirus	v-kit
X03711	Feline sarcoma virus (HZ4)	2473	Gammaretrovirus	v-kit
D88386	Friend murine leukemia virus	8358	Gammaretrovirus	-
M93134	Friend murine leukemia virus	8282	Gammaretrovirus	-
NC_001362	Friend murine leukemia virus	8323	Gammaretrovirus	-
AF030173	Friend spleen focus-forming virus	1935	Gammaretrovirus	-
J02193	Friend spleen focus-forming virus	1774	Gammaretrovirus	-
K00021	Friend spleen focus-forming virus, CG	6296	Gammaretrovirus	-
L27432	Friend spleen focus-forming virus, gp	1032	Gammaretrovirus	-
M20846	Friend spleen focus-forming virus, 3LTR	222	Gammaretrovirus	-
M90673	Friend spleen focus-forming virus, env	1071	Gammaretrovirus	-
V01552	Friend spleen focus-forming virus, gp52	1738	Gammaretrovirus	-
X02912	Friend spleen focus-forming virus, LTR	565	Gammaretrovirus	-
AF033810	Fujinami sarcoma virus	4788	Alpharetrovirus	v-FPS
X00188	Gardner-Arnstein feline leukemia oncovirus B	2611	Gammaretrovirus	v-fes
AF055061	Gibbon ape leukemia virus	2058	Gammaretrovirus	-
AF055062	Gibbon ape leukemia virus	2046	Gammaretrovirus	-
NC_001885	Gibbon ape leukemia virus	8088	Gammaretrovirus	-
U20589	Gibbon ape leukemia virus	2849	Gammaretrovirus	-
U60065	Gibbon ape leukemia virus	8379	Gammaretrovirus	-
J02207	Harvey murine sarcoma virus	997	Gammaretrovirus	v-rasH
Y07725	Human foamy virus	13242	Spumavirus	-
AY713411	Human immunodeficiency virus 1	8735	Lentivirus	-
U21247	Human spumaretrovirus	11954	Spumavirus	-
Y13051	Human T-cell lymphotropic virus type 2b	8960	Deltaretrovirus	-
X06391	Human T-cell lymphotropic virus type 4	5391	Lentivirus	-
AB513134	Human T-lymphotropic virus 1	9034	Deltaretrovirus	-

AF033817	Human T-lymphotropic virus 1	8507	Deltaretrovirus	-
AF042071	Human T-lymphotropic virus 1	8868	Deltaretrovirus	-
AF139170	Human T-lymphotropic virus 1	9031	Deltaretrovirus	-
AF259264	Human T-lymphotropic virus 1	9034	Deltaretrovirus	-
AY563954	Human T-lymphotropic virus 1	8883	Deltaretrovirus	-
HQ606137	Human T-lymphotropic virus 1	9039	Deltaretrovirus	-
HQ606138	Human T-lymphotropic virus 1	9039	Deltaretrovirus	-
J02029	Human T-lymphotropic virus 1	9068	Deltaretrovirus	-
L03561	Human T-lymphotropic virus 1	9043	Deltaretrovirus	-
U19949	Human T-lymphotropic virus 1	9036	Deltaretrovirus	-
AF074965	Human T-lymphotropic virus 2	8893	Deltaretrovirus	-
AF139382	Human T-lymphotropic virus 2	8958	Deltaretrovirus	-
AF326583	Human T-lymphotropic virus 2	8964	Deltaretrovirus	-
AF326584	Human T-lymphotropic virus 2	8955	Deltaretrovirus	-
AF412314	Human T-lymphotropic virus 2	8962	Deltaretrovirus	-
GU212854	Human T-lymphotropic virus 2	8953	Deltaretrovirus	-
L11456	Human T-lymphotropic virus 2	8956	Deltaretrovirus	-
M10060	Human T-lymphotropic virus 2	8952	Deltaretrovirus	-
DQ093792	Human T-lymphotropic virus 3	8917	Deltaretrovirus	-
DQ462191	Human T-lymphotropic virus 3	8553	Deltaretrovirus	-
EU649782	Human T-lymphotropic virus 3	8922	Deltaretrovirus	-
GQ463602	Human T-lymphotropic virus 3	8913	Deltaretrovirus	-
AF357971	Jaagsiekte sheep retrovirus	11791	Betaretrovirus	-
DQ838493	Jaagsiekte sheep retrovirus	7942	Betaretrovirus	-
DQ838494	Jaagsiekte sheep retrovirus	7430	Betaretrovirus	-
NC_001494	Jaagsiekte sheep retrovirus	7462	Betaretrovirus	-
Z23152	Kirsten murine sarcoma virus	1263	Gammaretrovirus	v-rasK
X54482	Macaque simian foamy virus	12972	Spumavirus	-
AF462057	Moloney murine leukemia virus	8332	Gammaretrovirus	-
NC_001501	Moloney murine leukemia virus	8332	Gammaretrovirus	-
J02266	Moloney murine sarcoma virus	5828	Gammaretrovirus	v-raf
K01691	Moloney murine sarcoma virus	1514	Gammaretrovirus	v-raf
V01184	Moloney murine sarcoma virus	4226	Gammaretrovirus	v-raf
X00740	Moloney murine sarcoma virus	1042	Gammaretrovirus	v-raf
AF033807	Mouse mammary tumor virus	8805	Betaretrovirus	-
K01683	Myeloproliferative sarcoma virus	2854	Gammaretrovirus	v-mpl
FJ744146	Ovine enzootic nasal tumor virus	7439	Betaretrovirus	-
FJ744147	Ovine enzootic nasal tumor virus	7436	Betaretrovirus	-
FJ744148	Ovine enzootic nasal tumor virus	7436	Betaretrovirus	-
FJ744150	Ovine enzootic nasal tumor virus	7425	Betaretrovirus	-
GU292314	Ovine enzootic nasal tumor virus	7436	Betaretrovirus	-
GU292317	Ovine enzootic nasal tumor virus	7439	Betaretrovirus	-
GU292318	Ovine enzootic nasal tumor virus	7439	Betaretrovirus	-
NC_007015	Ovine enzootic nasal tumor virus	7434	Betaretrovirus	-
K02375	Rauscher spleen focus-forming virus	2163	Gammaretrovirus	
J02342	Rous sarcoma virus - Prague C	9625	Alpharetrovirus	v-src
AF052428	Rous sarcoma virus - Schmidt-Ruppin B	9396	Alpharetrovirus	v-src
NC_001364	Simian foamy virus	13246	Spumavirus	-
M74895	Simian foamy virus 3	13111	Spumavirus	-
HM245790	Simian foamy virus-gorilla	12258	Spumavirus	-
AJ544579	Simian foamy virus-orangutan	12823	Spumavirus	-
GU356394	Squirrel monkey foamy virus	11684	Spumavirus	-
NC_001618	UR2 sarcoma virus	3166	Alpharetrovirus	v-ros
AF033822	Walleye dermal sarcoma virus	12708	Epsilonretrovirus	
EF428979	Walleye dermal sarcoma virus	12711	Epsilonretrovirus	
AF133051	Walleye epidermal hyperplasia virus 1	12999	Epsilonretrovirus	
AF133052	Walleye epidermal hyperplasia virus 2	13125	Epsilonretrovirus	
V01201	Woolly monkey sarcoma virus	5779	Gammaretrovirus	v-sis
NC_008094	Y73 sarcoma virus	5188	Alpharetrovirus	v-yes
V01170	Y73 sarcoma virus	3718	Alpharetrovirus	v-yes

Supplementary Table 2: Summary of sequencing of cells from HIV-1-infected individuals

Sample ID	Subtype	Viral load (RNA copies/ml)	Viral load (DNA copies/ml)	Method	Total reads	Human reads	Unique human reads	Subtype B unique reads*	Proportion of subtype B reads (ppm)*	Subtype A1 unique reads§	Proportion of subtype A1 reads (ppm)§
CGG_5_595	B	851,000	Unknown	Shotgun	67,359,234	64,023,339	62,045,309	0	0	0	0
CGG_5_595	B	851,000	Unknown	Capture	54,585,842	50,897,069	36,410,735	27	0.74	16	0.44
CGG_5_596	A	18,000	Unknown	Shotgun	78,385,281	74,755,675	72,718,879	0	0	0	0
CGG_5_596	A	18,000	Unknown	Capture	25,198,962	23,711,304	20,985,699	10	0.48	10	0.48

*Subtype B reference genome (HxB2), Genbank: K03455.1

§Subtype A1 reference genome (UG037), Genbank: AB253429

Supplementary Table 3: Summary of individual cancer samples

Sample ID	Disease	Dataset ID	NA	Method	Trimmed reads (No.)	Reads after hg19 digital subtraction (No.)	Reads mapping to virus reference genomes (Species)
CGG_5_000270	T-lymphoma	s329/s335/s341	DNA	Shotgun	209,798,998	4,696,528	Parvovirus B19
CGG_5_000272	T-lymphoma	s330/s336/s342	DNA	Shotgun	248,899,386	5,904,758	HHV-6
CGG_5_000274	T-lymphoma	s331/s337/s343	DNA	Shotgun	196,124,894	4,633,126	HHV-6A
CGG_5_000275	T-lymphoma	s332/s338/s344	DNA	Shotgun	187,520,102	4,125,528	HHV-6
CGG_5_000277	T-lymphoma	s333/s339/s345	DNA	Shotgun	173,991,074	4,189,896	HHV-6
CGG_5_000279	T-lymphoma	s334/s340/s346	DNA	Shotgun	131,790,372	3,117,772	
CGG_5_000270	T-lymphoma	s357/s363	DNA	Capture	151,545,878	2,916,218	Parvovirus B19
CGG_5_000272	T-lymphoma	s358/s364	DNA	Capture	131,828,100	2,054,504	
CGG_5_000274	T-lymphoma	s359/s365	DNA	Capture	131,382,178	2,525,660	HHV-6A
CGG_5_000275	T-lymphoma	s360/s366	DNA	Capture	118,816,020	2,021,672	
CGG_5_000277	T-lymphoma	s361/s367	DNA	Capture	191,065,524	5,232,724	
CGG_5_000279	T-lymphoma	s362/s368	DNA	Capture	150,580,320	3,092,258	
CGG_5_000091	B-lymphoma	s073	DNA	Shotgun	51,949,348	2,408,148	
CGG_5_000094	B-lymphoma	s074	DNA	Shotgun	108,737,616	4,434,762	
CGG_5_000096	B-lymphoma	s075	DNA	Shotgun	49,310,551	2,545,921	
CGG_5_000098	B-lymphoma	s076	DNA	Shotgun	137,345,602	6,103,100	
CGG_5_000100	B-lymphoma	s077	DNA	Shotgun	54,136,964	1,400,568	
CGG_5_000105	B-lymphoma	s078	DNA	Shotgun	72,279,874	1,814,702	
CGG_5_000107	B-lymphoma	s079	DNA	Shotgun	58,819,038	1,816,478	
CGG_5_000110	B-lymphoma	s080	DNA	Shotgun	55,983,060	1,404,008	
CGG_5_000117	B-lymphoma	s081	DNA	Shotgun	35,994,744	454,254	
CGG_5_000121	B-lymphoma	s083	DNA	Shotgun	30,486,372	1,018,976	
CGG_5_000123	B-lymphoma	s084	DNA	Shotgun	27,504,684	821,234	
CGG_5_000091	B-lymphoma	s015	DNA	Capture	61,535,876	1,885,400	
CGG_5_000094	B-lymphoma	s016	DNA	Capture	62,502,858	2,171,012	
CGG_5_000096	B-lymphoma	s017	DNA	Capture	59,101,898	1,727,348	
CGG_5_000098	B-lymphoma	s018	DNA	Capture	64,406,524	2,001,562	
CGG_5_000100	B-lymphoma	s019	DNA	Capture	54,812,690	2,128,430	
CGG_5_000105	B-lymphoma	s020	DNA	Capture	54,966,300	1,868,978	
CGG_5_000107	B-lymphoma	s021	DNA	Capture	51,195,158	2,560,194	
CGG_5_000110	B-lymphoma	s022	DNA	Capture	62,382,580	2,078,768	
CGG_5_000117	B-lymphoma	s023	DNA	Capture	48,200,078	1,623,340	
CGG_5_000119	B-lymphoma	s024	DNA	Capture	55,469,702	2,445,462	
CGG_5_000121	B-lymphoma	s025	DNA	Capture	53,498,356	1,984,022	
CGG_5_000123	B-lymphoma	s026	DNA	Capture	56,252,604	980,630	
CGG_5_000092	B-lymphoma	s027	RNA	Shotgun	100,089,878	9,396,326	PHV, ALV, EBV
CGG_5_000099	B-lymphoma	s028	RNA	Shotgun	92,436,654	9,200,122	PHV, ALV
CGG_5_000118	B-lymphoma	s030	RNA	Shotgun	117,654,754	8,801,484	PHV, ALV,EBV
CGG_5_000119	B-lymphoma	s035	RNA	Shotgun	96,790,430	8,068,990	PHV, ALV
CGG_5_000122	B-lymphoma	s036	RNA	Shotgun	109,303,544	11,133,196	PHV, ALV
CGG_5_000092	B-lymphoma	s038	RNA	Capture	58,741,836	9,644,744	ALV, PHV
CGG_5_000099	B-lymphoma	s039	RNA	Capture	74,264,728	12,962,362	ALV, PHV
CGG_5_000118	B-lymphoma	s040	RNA	Capture	82,090,514	14,115,944	ALV, PHV
CGG_5_000120	B-lymphoma	s041	RNA	Capture	63,264,246	9,601,804	ALV, PHV
CGG_5_000122	B-lymphoma	s042	RNA	Capture	51,482,778	11,408,426	ALV, PHV
CGG_5_000124	B-lymphoma	s043	RNA	Capture	57,451,190	8,109,208	ALV, PHV
CGG_5_000004	Colon cancer	s094	DNA	Shotgun	369,901,822	6,865,963	
CGG_5_000007	Colon cancer	s005	DNA	Shotgun	40,572,942	1,389,074	
CGG_5_000017	Colon cancer	s004	DNA	Shotgun	55,611,513	674,636	
CGG_5_000029	Colon cancer	s008	DNA	Shotgun	46,096,566	786,114	
CGG_5_000034	Colon cancer	s007	DNA	Shotgun	33,190,713	456,074	
CGG_5_000038	Colon cancer	s006	DNA	Shotgun	34,655,026	472,995	
CGG_5_000042	Colon cancer	s003	DNA	Shotgun	45,929,779	613,427	
CGG_5_000047	Colon cancer	s095	DNA	Shotgun	170,931,578	6,399,600	
CGG_5_000053	Colon cancer	s096	DNA	Shotgun	202,785,130	6,034,833	
CGG_5_000056	Colon cancer	s097	DNA	Shotgun	195,020,756	3,064,682	
CGG_5_000061	Colon cancer	s098	DNA	Shotgun	191,605,216	4,483,613	Adenovirus
CGG_5_000068	Colon cancer	s099	DNA	Shotgun	168,653,677	4,041,606	
CGG_5_000071	Colon cancer	s100	DNA	Shotgun	187,202,791	3,481,688	PHV, HHV-6
CGG_5_000007	Colon cancer	s014	DNA	Capture	34,187,715	1,401,500	
CGG_5_000017	Colon cancer	s013	DNA	Capture	81,785,286	1,761,298	
CGG_5_000029	Colon cancer	s011	DNA	Capture	77,616,532	2,281,222	
CGG_5_000034	Colon cancer	s010	DNA	Capture	54,810,626	1,212,200	
CGG_5_000038	Colon cancer	s009	DNA	Capture	75,303,740	1,817,404	
CGG_5_000042	Colon cancer	s012	DNA	Capture	73,760,542	1,576,962	HHV-5
CGG_5_000033	Colon cancer	s288	RNA	Shotgun	70,705,002	2,134,250	ALV, PHV
CGG_5_000037	Colon cancer	s289	RNA	Shotgun	7,236,478	713,446	ALV, PHV
CGG_5_000043	Colon cancer	s290	RNA	Shotgun	10,103,908	438,898	ALV, PHV
CGG_5_000049	Colon cancer	s291	RNA	Shotgun	84,209,784	3,053,426	ALV, PHV
CGG_5_000055	Colon cancer	s292	RNA	Shotgun	116,430,750	3,942,464	ALV, PHV
CGG_5_000058	Colon cancer	s293	RNA	Shotgun	77,700,308	3,306,666	ALV, PHV
CGG_5_000063	Colon cancer	s294	RNA	Shotgun	93,171,602	5,623,960	ALV, PHV
CGG_5_000069	Colon cancer	s295	RNA	Shotgun	71,385,952	10,616,684	ALV, PHV
CGG_5_000073	Colon cancer	s296	RNA	Shotgun	102,725,966	4,744,962	ALV, PHV
CGG_5_000078	Colon cancer	s297	RNA	Shotgun	11,554,106	1,830,812	ALV, PHV
CGG_5_000084	Colon cancer	s298	RNA	Shotgun	63,744,248	3,760,296	ALV, PHV

ALV: Avian Leucosis virus. PHV parvovirus-like hybrid virus. HHV: Human herpesvirus. EBV: Ebstein-Barr virus.

Supplementary Table 4: Primers and probes for qPCR analysis

**Oligo ID**	**Sequence 3' – 5'**	**5'-dye, 3'-quencher**
Bx08gagF	AGGCCAGGGAATTTTCTTCAG	
Bx08gagR	GGAGTTGTTGCTTCCCCAAA	
Bx08gagP	AGACCAGAGCCAACAGCCCCACC	FAM, BHQ1
B2m_LVIF1	CAAATCCCACTGTCACATGCA	
B2m_LVIR1	TGGTTGAGTTGGACCCGATAA	
B2m_LVIP1	TCCCATTTGCCATAGTCCTCACCTATCCCT	HEX, BHQ1

Supplementary Figure S1

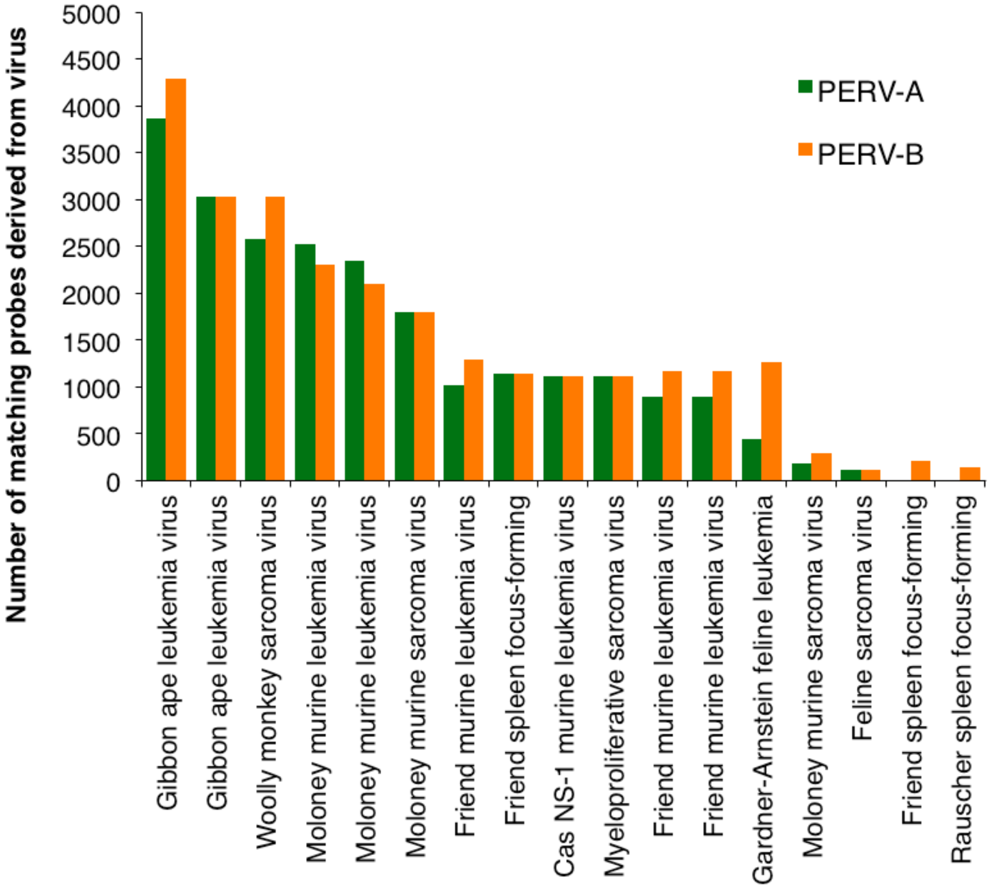

Supplementary Figure S1: Capture probes with similarity to PERV-A or PERV-B. The numbers are shown of matching probes derived from each reference genomes.

Supplementary Figure S2

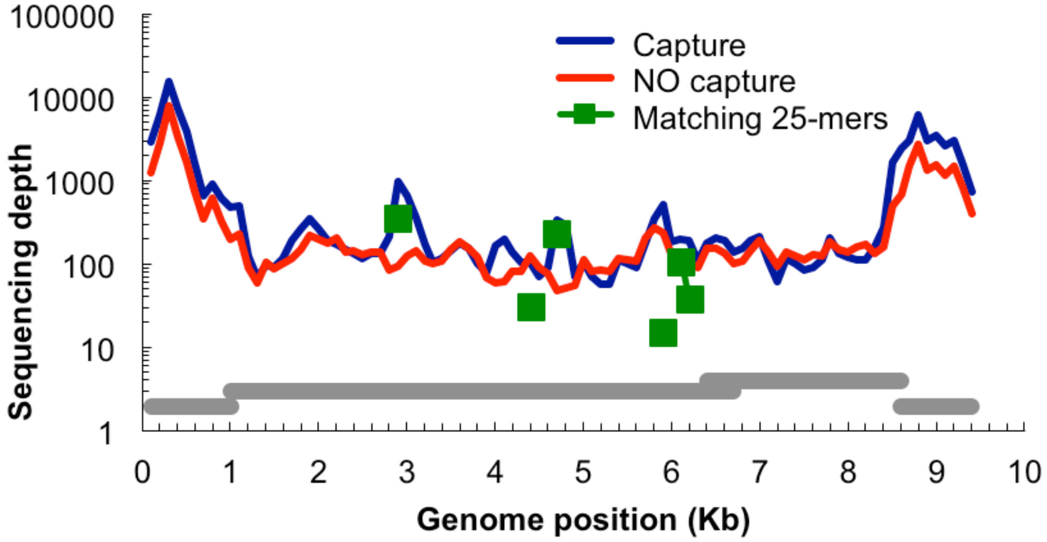

Supplementary Figure S2: Distribution of capture probes with similarity to HERV-K (AY037928). The sequencing depth obtained over the genome is shown from captured or non-captured material in blue and red, respectively. Below, the approximate genomic organisation (LTR-gag-pol-env-LTR) of HERV-K is indicated in grey.
